# Vaccinnation Objection

**Published:** 1872-11

**Authors:** 


### Vaccinnation Objection.

A small boy in an Indiana town made several applications to be protected against the scourge of small-pox. Finally his physician was able to accommodate him with an excellent "scab" from a healthy boy; but when the patient found where it came from, he objected to the application being made for fear he would "stutter," as the boy who furnished the material was addicted to that method of articulating.

